# Genome-wide identification of PME gene family and expression of candidate genes associated with aluminum tolerance in tea plant (*Camellia sinensis*)

**DOI:** 10.1186/s12870-022-03686-7

**Published:** 2022-06-24

**Authors:** Danjuan Huang, Yingxin Mao, Guiyi Guo, Dejiang Ni, Liang Chen

**Affiliations:** 1 Tea Research Institute, Chinese Academy of Agricultural Sciences, Hangzhou, 310008 China; 2 Fruit and Tea Research Institute, Hubei Academy of Agricultural Sciences, Wuhan, 430064 China; 3 College of Horticulture and Forestry Science, Huazhong Agricultural University, Wuhan, 430070 China; 4 Henan Key Laboratory of Tea Plant Comprehensive Utilization in South Henan, Xinyang Agriculture and Forestry University, Xinyang, 464000 China

**Keywords:** Pectin methylesterase, Aluminum tolerance, Expression levels, Tea plant

## Abstract

**Background:**

The major aluminum (Al) detoxication mechanism of tea plant (*Camellia sinensis*), as an Al hyperaccumulator plant, is the fixation of almost 70% of Al in the cell walls. Pectin is the primary constituent of cell walls, a degree of methylation of pectin polysaccharides regulated by the pectin methylesterase (PME) genes can greatly affect the Al binding capacity. The knowledge on *PME* gene family in tea plant is still poor.

**Results:**

We identified 66 (*CsPME1-CsPME66*) *PME* genes from *C. sinensis* genome. We studied their protein characterization, conserved motifs, gene structure, systematic evolution and gene expression under Al treatments, to establish a basis for in-depth research on the function of *PMEs* in tea plant. Gene structures analysis revealed that the majority of *PME* genes had 2–4 exons. Phylogenetic results pointed out that the *PME* genes from the same species displayed comparatively high sequence consistency and genetic similarity. Selective pressure investigation suggested that the Ka/Ks value for homologous genes of *PME* family was less than one. The expression of *CsPMEs* under three Al concentration treatments was tissue specific, eight *PME* genes in leaves and 15 in roots displayed a trend similar to of the Al contents and PME activities under Al concentration treatments, indicating that the degree of pectin de-esterification regulated by PME was crucial for Al tolerance of tea plant.

**Conclusions:**

Sixty-six *CsPME* genes were identified for the first time in tea plant. The genome-wide identification, classification, evolutionary and transcription analyses of the *PME* gene family provided a new direction for further research on the function of *PME* gene in Al tolerance of tea plant.

**Supplementary Information:**

The online version contains supplementary material available at 10.1186/s12870-022-03686-7.

## Background

In highly acidic soils, soluble aluminum (Al) is a primary restraint on plant growth because it inhibits root elongation. As these soils represent nearly half of the arable land in the world, Al toxicity is a significant problem in agriculture. Being an Al-accumulating crop, tea plants contain large amounts of Al in both leaves and roots without exhibiting the Al toxicity symptoms. The amount of Al accumulated in tea plant was much higher than in the other plant species cultivated in the same location, the Al content in old leaves of tea plant ranged from 1350 to 2100 mg/kg compared with leafy vegetables (from 200 to 650 mg/kg) and grains containing so little Al it was almost undetectable [1]. Subcellular distributions analysis demonstrated that 75.2 and 69.8% of the total Al were detected in the cell walls of tea leaves and roots, respectively [2]. The cell wall was regarded as a physical barrier protecting the internal plant cell structures from Al toxicity. Therefore, the components, structure and modifications of the cell wall are important for the Al stress responses.

Plant cell wall is principally composed of pectin, cellulose and hemicellulose. Of them, the de-methylesterification of the homogalacturonan (HG) component of pectin is catalyzed by pectin methylesterases (PMEs) [3]. PMEs influence the cell wall in two different ways: (i) the combination of negatively charged carboxyl groups with extracellular free Ca^2+^ results in the regular pectin arrangement, hence stiffening the cell wall and slowing cell growth [4] and (ii) PMEs catalyse the production of free carboxyl groups from HG and the release of protons, thus lowering the extracellular pH enhancing the activities of hydrolytic enzymes, including polygalacturonase (PG) and pectinlyase (PL). Consequently, pectin undergoes extensive degradation, giving rise to the cell wall loosening and expansion, and then accelerated growth of cell tips [5]. In plants, *PMEs* belong to a large multigene family and involved in various growth and developmental processes, including root development, stem elongation, pollen tube germination, fruit ripening, as well as plant stress responses [4, 6]. Up to now, 66 *PMEs* in *Arabidopsis* [7], 43 in rice [8], 105 in flax [9], and 80 in *Gossypium arboreum* [10] had been reported. Increasing experimental evidence has indicated the degree of methyl modification in pectin polysaccharides can greatly influence the Al binding capacity of pectin polysaccharides. For instance, Schmohl et al. and Horst et al. reported that the activity of PME was associated with Al resistance and Al-induced inhibition of root elongation [11, 12]. In rice, the Al-sensitive cultivar showed constitutively higher PME activity in root tip cell wall than the Al resistant cultivar. The expression profiles of 35 *PME* genes suggested that eight *PME* genes were up-regulated after 25 μM Al treatment. Among these eight *PME* genes, overexpressing of *OsPME14* in transgenic rice increased sensitivity to Al toxicity [13, 14]. These results adequately demonstrated the role of *PME* in regulating plant Al resistance.

There are reports that *PMEs* play significant roles in Al tolerance in tea plant via involvement in the cell wall development [15, 16]. However, the comprehensive analysis of *PME* genes at the whole genome level does not appear to have been reported. In 2020, several chromosome-level genomes were completed, which greatly improved the degree of accuracy and integrity of genome assembly [17–21]. Furthermore, haplotype-resolved genome of tea plant became available in 2021. The completion of high-quality whole genome sequencing of tea plant offers the chance for synthetic study of the *PME* gene family. In this study, we performed a thorough analysis of *CsPMEs* in tea plant on the basis of the whole genome sequence of *C. sinensis* [17]. We also carried out a comprehensive study of all the *CsPMEs* at the transcriptional level under three Al concentration treatments. Our research sheds light on the molecular functions of *CsPMEs* under Al stress.

## Materials and methods

### Materials and processing methods

Six-month-old plug seedlings of *C. sinensis* ‘E’Cha 1’ were grown in a greenhouse of Fruit and Tea Research Institute, Hubei Academy of Agricultural Sciences, Wuhan, China. The plug seedlings were rinsed thoroughly with pure water, and then transplanted in incubators (245 mm × 170 mm × 75 mm) containing 3 kg of sterilized sand. Plants (six per pot, three pots per treatment) were irrigated with nutrient solutions (pH 5.0, 400 mL per pot), with three different levels (0, 1, and 4 mM) according to the previous study [16]. The plants were cultivated in a phytotron with day/night of 12/12 h, photon flux density of 300–400 μmol m^2^s^− 1^, and relative humidity of 70%; temperature was maintained at 24 °C. After 1 week of Al treatments, soft roots and the second leaves were harvested. In addition, apical bud, young leaves, mature leaves, old leaves, stem, roots, flowers, and fruits were harvested during the development stage of 5-year-old ‘E’cha 1’ plants. All samples were snap frozen in liquid nitrogen and stored at − 80 °C.

### Al content and PME activity measurements

An amount of 0.4 g of fresh weight of leaf sample powder was digested in a mixture of HNO_3_:HClO_4_ (5:2; v/v), adjusted to 50 mL with distilled water, and filtered through a 0.45-μm organic membrane before Al was analyzed by an inductively coupled plasma optical emission spectrometer (ICP-OES). The PME activity was determined by using a Pectinesterase reagent kit® (Qiyi, Shanghai, China) according to a manufacturer’s instructions. The measurements were repeated thrice, and the average values ± standard error were calculated.

### RNA isolation and qRT-PCR

The extraction of total RNA was conducted using a Polysaccharide Polyphenol RNA Extraction Kit® (Simgen, Hangzhou, China), based on the standard protocols. Then, 1 μg RNA was used for the synthesis of the first strand cDNA by using the cDNA First Strand Synthesis Kit^®^ (Simgen, Hangzhou, China). Real-time qPCR was performed using 2 × SYBR^®^ Green PCR Mix (Simgen, Hangzhou, China) and conducted on Applied Biosystem™ 7500 (Thermo Fisher Scientific, USA) with the following protocol: 95 °C for 30 s; 40 cycles of 95 °C for 5 s each, annealing at 60 °C for 34 s; 95 °C for 15 s, 60 °C for 1 min, and 95 °C for 15 s. Three biological and three technical replicates were performed for every treatment. The relative expression level was calculated by the 2^−ΔΔCt^ method [22]. The internal reference gene was *CsGAPDH*. The primer sequences of *CsPME*s are listed in Additional file 1 Table. S5. The gene expression heatmaps were drawn using TBtools [23].

### Identification of the PME family

The HMM (Hidden Markov Model) profiles of PME domain (PF01095) and PMEI (pectin methyl esterase inhibitor) domain (PF04043) were obtained from the PFam database (http://pfam.sanger.ac.uk/), and the HMMER software [24] was applied to search for *PME* genes in tea plant genome ‘Shuchazao’ with the best domain e-value cutoff of 1e^− 100^. The presence of an entire PME domain was checked for all potential PME proteins by SMARAT (http://smart.embl-heidelberg.de/). Then, we used HMM profiles of PF04043 (PMEI domain) to search in the PME protein sequences to detect *PME* genes that containing the PMEI domain. Predicted tea proteins which had both PME and PMEI domain were denoted type-1 *PMEs*, and proteins with only the PME domain were labeled type-2 *PMEs*.

### Chromosomal location and gene duplication analysis

The gene ID of *CsPMEs* were used to search in tea plant (*C.sinensis*) gff file downloaded from http://tpdb.shengxin.ren/ for gaining chromosomal location information. Next, we detected gene duplication events of *CsPMEs* based on E-value ≤1e-5, identity> 70%. The Circos-0.69 Software was used to visualize chromosomal location and gene duplication [25]. Then, MCscanX was employed to identify homologous regions [26]. The KaKs Caculator2.0 was used to calculate the Ka/Ks (The ratio of the number of nonsynonymous substitutions per nonsynonymous site (Ka) to the number of synonymous substitutions per synonymous site (Ks)) value of these homologous genes [27].

### Protein characterization analysis of PMEs family

The ExPASy ProtParam tool (https://web.expasy.org/protparam/) was applied to evaluate the properties of *CsPME* proteins, including the number of amino acids, molecular weight (MW), theoretical isoelectric point (pI), grand average of hydropathicity (GRAVY), and so on. We predicted the subcellular localization via both Cell-PLoc 2.0 (http://www.csbio.sjtu.edu.cn/bioinf/Cell-PLoc-2/) and WOLF PSORT ProtParam tools (https://wolfpsort.hgc.jp/). In addition, the signal peptides were predicted through the SignalP 4.1 Server (https://services.healthtech.dtu.dk/service.php?SignalP-5.0). The transmembrane regions were predicted using the TMHMM Server v.2.0 (https://services.healthtech.dtu.dk/service.php?TMHMM-2.0).

### Gene structure and motif analysis and promoter analyses

The Gene Structure Display Server (http://gsds.gao-lab.org/) was used to determine the distribution characteristics of exons and introns [28]. The conserved motifs were searched for by software MEME v5.3.3 [29]. The 2.0 kb DNA upstream sequences of ATG were extracted to predict the cis-acting regulatory elements using PlantCARE (http://bioinformatics.psb.ugent.be/webtools/plantcare/html/) [30].

### Phylogenetic analysis

For identification of PME proteins in *Populus trichocarpa* (black cottonwood), *Coffea arabica* (coffee), *Theobroma cacao* (cacao), and *Actinidia chinensis* (kiwifruit), we used the HMM profiles of PF01095 (PME domain) to search in the protein sequences downloaded from https://www.ncbi.nlm.nih.gov/genome/?term=. In addition, 47 PME proteins of *Vitis vinifera* (grapevine) [31] were retrieved from Ensembl (https://plants.ensembl.org/index.html), 66 putative PME proteins of *Arabidopsis* [7] were obtained from the TAIR (http://www.arabidopsis.org/browse/genefamily/), and 43 rice PME protein sequences [8] were extracted from the rice genome at https://www.ncbi.nlm.nih.gov/genome/?term=RICE. Then, all of the PME protein sequences from seven species were aligned with the PME protein sequences of tea plant by ClustalW within the MEGA 7.0 software package. A phylogenetic tree of the aligned sequences was constructed in MEGA 7.0 [32] by the neighbor-joining method with Poisson distribution, pairwise deletion and bootstrap values of 1000 and was visualized in iTOL (https://itol.embl.de/) [33].

## Results

### Identification of tea *PMEs*

**Fig. 1:**
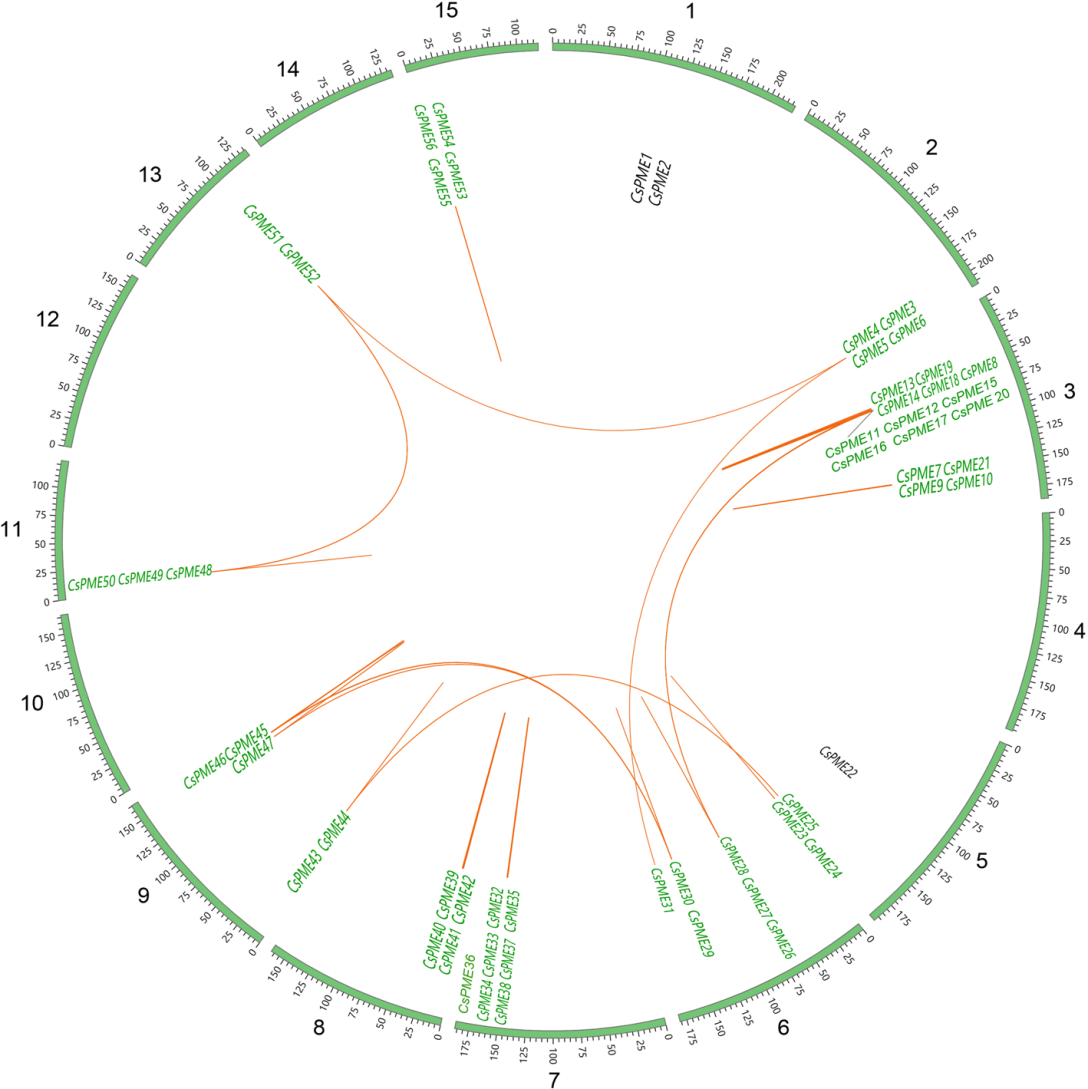
Chromosomal distribution and collinear correlation of *PME* genes in tea plant. The scale is in megabases (Mb), the value on each chromosome represents chromosome length, the paralogous *CsPMEs* are highlighted with green color and connected with a red line

To identify putative PME proteins in tea plant, we searched for proteins within PME (PF01095) and PMEI (PF04043) domains and obtained 66 *CsPMEs*. Based on their locations and ordering on the chromosomes, we named the family members from *CsPME1* to *CsPME66* (Table S1; Fig. 1a). Among the 66 *PMEs*, 58 tea *PMEs* (88%) were identified as type-1 *PMEs* (proteins containing both PME and PMEI domains) and eight (12%) were identified as type-2 *PMEs* (proteins contain only PME domain).

### Chromosomal location and gene duplication of *CsPMEs*

Fifty-six genes were located on 11 chromosomes, showing an uneven distribution (Fig. 1). The largest (15) number of *PMEs* was located on chromosome 3, followed by seven *PMEs* on chromosome 7. Six *PME* genes were positioned on chromosome 6, five on chromosome 9, four genes on chromosomes 2, 5, 8 and 15, three on chromosome 11, and two on chromosomes 1 and 13. Ten genes were not found on the chromosomes but were anchored on the scaffolds instead.

The duplication events may illuminate the mechanism about the expansion of *CsPME* gene family. Therefore, we detected the gene pairs in *CsPME* family. A total of 120 gene pairs were detected in *CsPME* gene family, and some genes repeatedly participate in gene duplication events (Fig. 1). In these gene pairs, 68 paralogous genes are located on the same chromosomes, suggesting that tandem duplication is the primary expansion model of tea *PME* gene family. On the contrary, 52 pairs are distributed on diverse chromosomes, indicating that segmental duplication also contributes to the expansion of *CsPME* family.

The Ka/Ks value can be regarded as an indicator of the selection pressure of a gene family in the process of evolutional history. To determine the selection influence on the evolution of the *PMEs* in tea plant, we calculate the Ka/Ks values of all the homologous genes (Additional file 1 Table. S4). Our results suggested that the Ka/Ks values were all < 1, which means that the *CsPMEs* were primarily determined by stabilizing selection.

### Protein characterization of PMEs family members

The CDS (coding sequences) ranged from 669 (*CsPME3*) to 3624 bp (*CsPME25*) with theoretical proteins of 222–1207 amino acids and MW from 24.51 to 133.42 kD. The pIs were between 4.75 (*CsPME5*) and 9.65 (*CsPME10*). The GRAVY values of the 66 PME proteins were less than zero, suggesting they were all hydrophilic. The subcellular localization was determined via both WoLF PSORT and Plant-mPLoc. Based on Wolf PSORT, the majority of *CsPMEs* were located in the chloroplast, with the remainder in vacuole, mitochondria and cytoplasm. However, Plant-mPLoc predicted that all the *CsPMEs* were positioned in the cell wall. Thirty-nine *CsPMEs* (59%) were predicted to contain signal peptides. In addition, 23 *CsPMEs* (34.8%) were predicted to contain 1 TMH.

### Gene structures and conserved protein motifs of *CsPMEs*

**Fig. 2:**
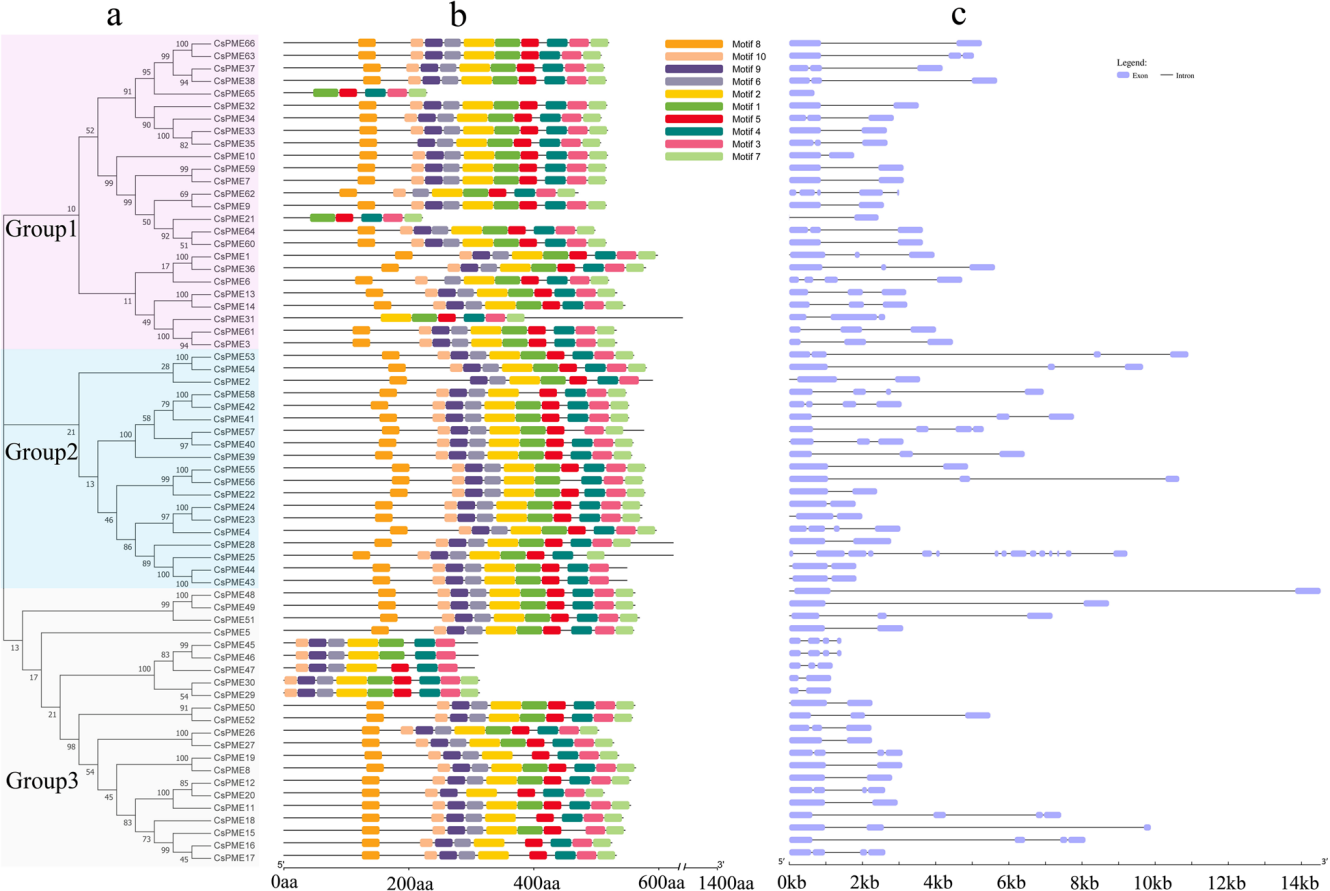
Phylogenetic tree(**a**), conserved motifs(**b**) and exon-intron structures(**c**) of the *CsPME*s. **a**. The three subfamilies are numbered as group 1 to 3; **b**. Motif composition of 66 *CsPMEs*, with different colors representing 10 common motifs; **c**. Exon-intron structures of *CsPME*s. Black lines denote introns, and blue boxes represent exons

Sixty-six members of *CsPMEs* in *C. sinensis* were categorized into three groups (Fig. 2a). Based on the sequence characteristics of *CsPME*s, we predicted 10 conserved motifs by MEME (Fig. 2b; Fig. S1). As shown in Fig. 2b, 41 *CsPMEs* (62.1%) contained all 10 motifs. Among the eight type-2 *CsPMEs* (proteins with only the PME domain), *CsPME21*, *CsPME31*, and *CsPME65* shared motifs 1, 3, 4, 5, and 7, and were classified in group 1. *CsPME29*, *CsPME30*, *CsPME45*, *CsPME46*, and *CsPME47* shared motifs 2, 3, 4, 6, 9 and 10, and were distributed in group 3. Each of them lacked motif 8.

To investigate the gene structure differences, the exon-intron distribution of *CsPMEs* was drawn by GSDS v2.0. As shown in Fig. 2c. The number of exons in 63 *CsPME* genes (account for 95% of all *CsPMEs*) ranged from two to four. The other three were *CsPME65*, *CsPME62,* and *CsPME25,* and they contained 1, 5 and 15 exons, respectively (Fig. 2c). In addition, *CsPME48* contained the longest intron (nearly 13 kb).

### Phylogenetic analysis

**Fig. 3:**
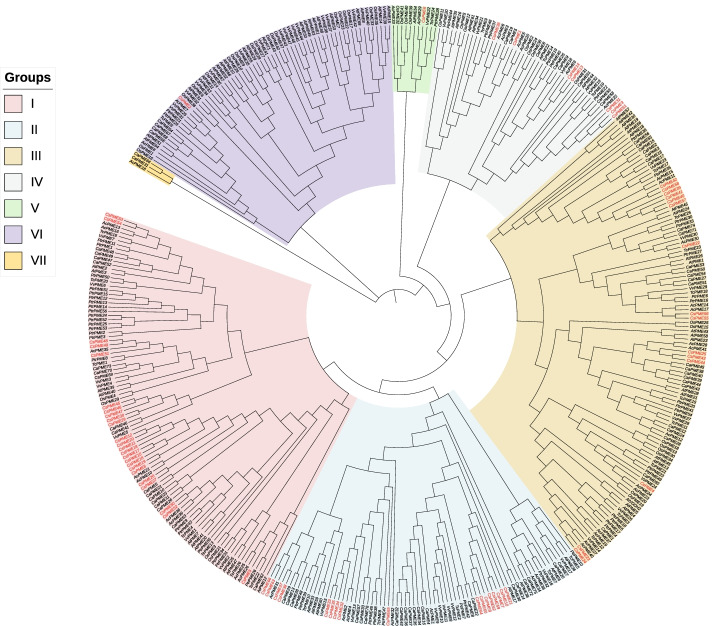
Phylogenetic analysis of 421 *PMEs* in tea plant, black cottonwood, coffee, cacao, kiwi fruit, grapevine, *Arabidopsis* and rice. *CsPMEs* were highlighted with red color. The seven groups were covered with different colors

According to the amino acid sequences, we analyzed the genetic evolution relationships of PME proteins by aligning the 66 *CsPMEs* with 56 from black cottonwood (*PtrPME*), 76 from coffee (*CaPME*), 26 from cacao (*TcPME*), 41 from kiwi fruit (*AcPME*), 47 from grapevine (*VvPME*), 66 from *Arabidopsis* (*AtPME*) and 43 from rice (*OsPME*) [7, 8]. As shown in Fig. 3, results revealed that tea *PMEs* were likely to be more correlated with kiwifruit proteins than those from other species. In addition, they clustered closely with each other in small branches of the evolutionary tree, implying they may perform new functions in tea plant. All *PME* genes were classified into seven major subfamilies (labeled as groups I to VII). The *CsPME* proteins were unevenly distributed, with 24 tea *PMEs* belonging to group I, 17 to group II, and 16 to group III (collectively representing 86.4% of all *CsPMEs*), with the remainder distributed as 7,1 and 1 in groups IV, V and VI, respectively. No *CsPMEs* belonged to group VII. In addition, group I-V comprised 350 *PMEs*, whereby 329 (94%) of them were type-1 *PMEs* containing both PME and PMEI domains. By contrast, groups VI and VII contained 71 *PMEs*, with 47(66.2%) of them being type-2 *PMEs*, consisting of only the PME domain. It is remarkable that all the *PMEs* from group VII were type-2.

### Analysis of *cis*-regulatory element distribution in *CsPME* promoters

**Fig. 4:**
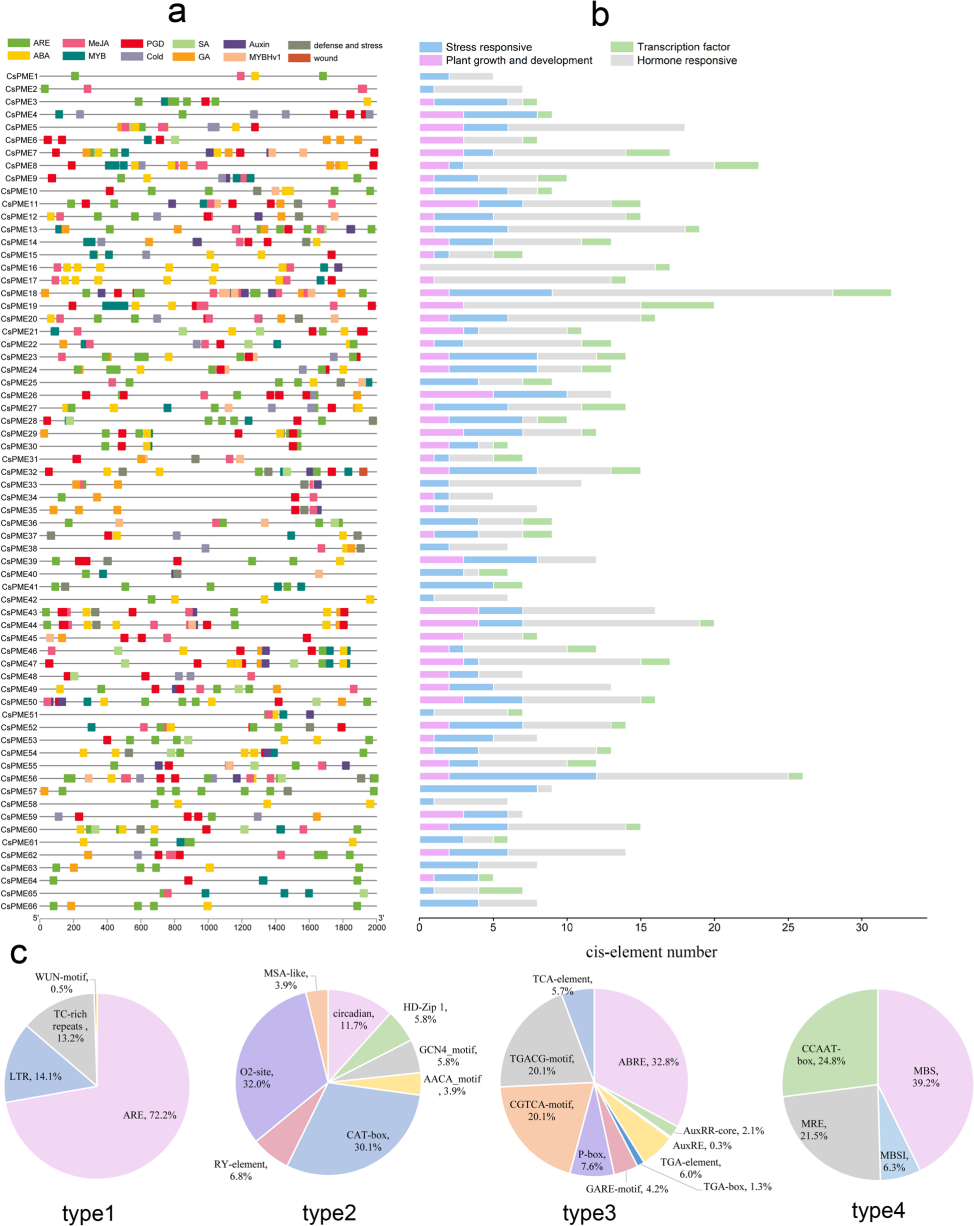
Analysis of *cis*-regulatory element distribution in the *CsPME* promoters **a**. The *cis*-elements in the *CsPME*s were marked by different colors. **b**. The number of *cis*-elements numbers in four categories; blue histograms (type1), stress responsive; purple histograms (type 2), plant growth and development; grey histograms (type 3), hormone responsive; and green histograms (type 4), transcription factor. **c**. The proportions of distinct *cis*-elements from four categories

To explore the regulatory network governing the *CsPME*s roles in the stress and growth responses, the *cis*-elements in the 2 kb 5′-upstream region of 66 *CsPMEs* were analyzed (Fig. 4a). We classified them into four categories, including stress responsive, plant growth and development, hormone responsive and transcription factor (Fig. 4b). Four types of *cis*-elements were identified in the stress responsive category, including ARE (anaerobic induction element), LTR (low-temperature responsiveness), TC-rich repeats, and WUN-motif (wound-responsive element). The ARE constituted the largest proportion (72.2%) of this category, followed by LTR (14.1%), TC-rich repeats (13.2%), and WUN-motif (0.5%). It is worth mentioning that WUN-motif was found only in *CsPME32*. In the category of plant growth and development, eight types of *cis*-elements were detected, which were CAT-box (meristem expression element), O2-site (zein metabolism regulation element), circadian control element, HD-Zip 1(element involved in differentiation of the palisade mesophyll cells), GCN4_motif (endosperm expression element), AACA_ motif (endosperm-specific negative expression), RY-element (seed-specific regulation element) and MSA-like (cell cycle regulation element). The O2-site accounted for the largest part (32.0%) of this category, followed by CAT-box (30.1%) and circadian (11.7%). In the hormone responsive category, 10 *cis*-element types were found, including CGTCA-motif and TGACG-motif responsive to MeJA (jasmonic acid methyl ester, 40.1%), ABRE related to ABA (abscisic acid) responsiveness (32.8%), P-box and GARE-motif referred to gibberellin (GA) responsiveness (11.7%), TCA-element responsive to salicylic acid (SA), and four types of cis-elements (AuxRR-core, AuxRE, TGA-element and TGA-box) related to auxin responsiveness. In the fourth category (transcription factor), there were three types of cis-element determined as MBS (MYB binding site responsive to drought inducibility, 39.2%), MRE (MYB binding site involved in light responsiveness, 21.5%), and MBSI (MYB binding site involved in flavonoid biosynthetic genes regulation, 6.3%). In addition, CCAAT-box was found to be the binding site of transcription factor MYBHv1, representing 24.8% of the fourth category (Fig. 4c), and numerous light responsive elements were found in all of the promoters of *CsPMEs* (data not shown).

### Expression levels of *CsPME* genes in different tissues

**Fig. 5:**
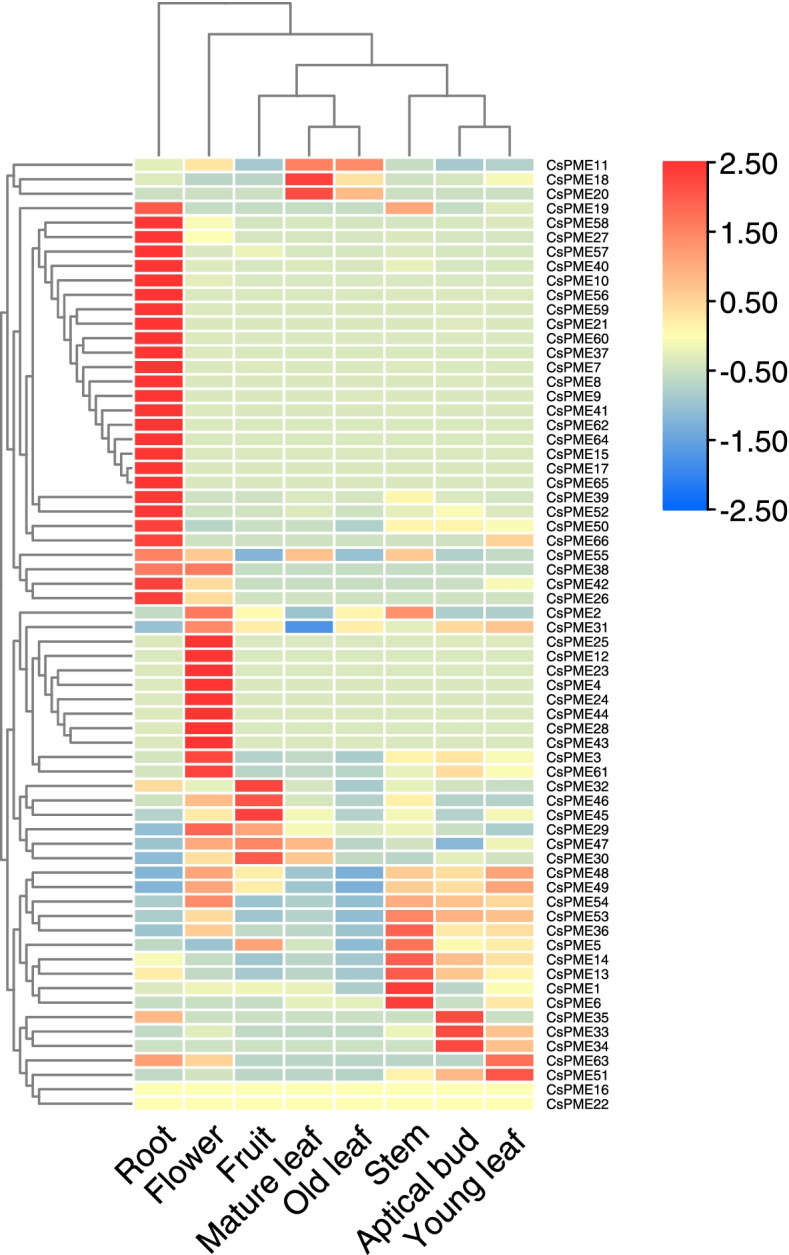
Expression levels of *CsPME* genes in different tissues. The normalized expression values are represented by a color scale histogram

As illustrated in Fig. 5, increased transcription (values > 2) was shown for 26 *CsPMEs* in roots and for 10 *CsPMEs* in flowers, but they had no or very low expression in other seven tissues. The *CsPME33, CsPME34* and *CsPME35* were highly expressed only in apical bud. The *CsPME51* was significantly expressed in young leaves. In addition, *CsPME 16* and *CsPME22* were not expressed in any of the eight tissues. In summary, the patterns of the *CsPME* genes expression varied in different tissues, suggesting a degree of tissue specificity.

### Al content, PME activity and expression patterns of *CsPME*s at different Al concentrations

**Fig. 6:**
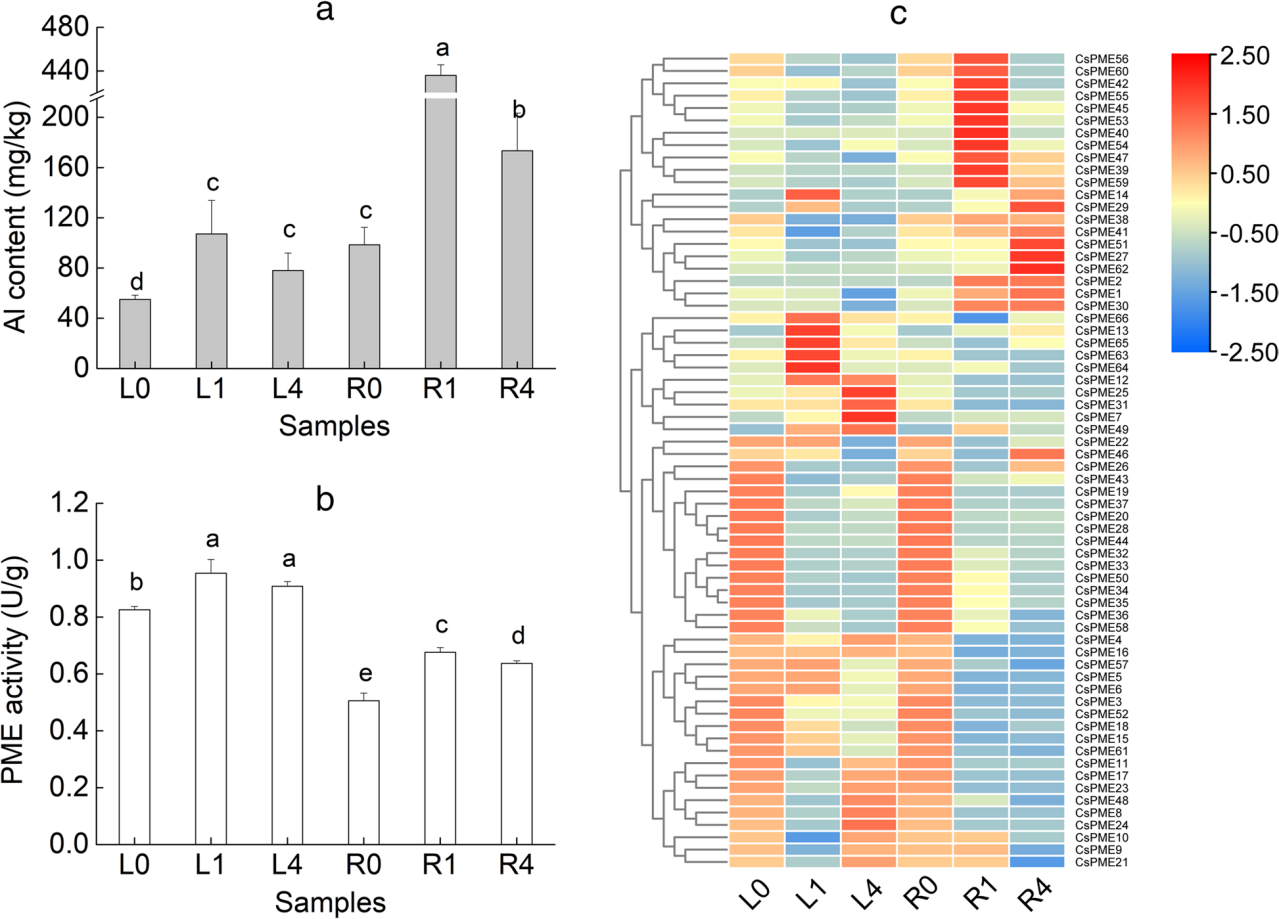
Al contents(a), PME activities(b) and transcriptional level of *CsPME* genes(c) under different Al treatments. L0(R0), L1 (R1) and L4(R4) mean leaf (root) samples under Al concentration treatments of 0, 1 and 4 mM, respectively. Values with the same letters are not significantly different (at α = 0.05) from each other. The normalized expression values are represented by a color scale histogram

As shown in Fig. 6a and b, the Al contents showed a similar trend with PME activities in both roots and leaves. Nevertheless, the trends in these two tissues were different. In leaves, the Al contents and PME activities both increased under 1 and 4 mM Al concentration treatments, with no significance difference between them. However, in roots, the Al contents and PME activities showed significant differences among 0, 1, and 4 mM Al concentration treatments, with a rise at 1 mM, and then a drop at 4 mM. The expression levels of *CsPME* genes in roots and leaves were altered in different Al treatments (Fig. 6c). In leaves, eight genes (*CsPME12, CsPME13, CsPME14, CsPME29, CsPME63, CsPME64, CsPME65,* and *CsPME66,*) were up-regulated at 1 mM Al treatment, but were down-regulated at 4 mM, exhibited the same trends as Al contents and PME activities. Nine genes (*CsPME8, CsPME9, CsPME10, CsPME11, CsPME17, CsPME21, CsPME23, CsPME24,* and *CsPME48*) were down-regulated in the leaves at 1 mM Al treatment, but upregulated at both 0 and 4 mM Al. In addition, the expression of five genes (*CsPME4, CsPME7, CsPME25, CsPME31,* and *CsPME49*) exhibited a rising trend with an increase of Al concentration. Similarly in roots, 15 genes (*CsPME1, CsPME2, CsPME30, CsPME39, CsPME40, CsPME42, CsPME45, CsPME47, CsPME49, CsPME53, CsPME54, CsPME55, CsPME56, CsPME59,* and *CsPME60*) were up-regulated at 1 mM Al treatment but were down-regulated at 4 mM, coinciding with the changes in Al contents and PME activities. Significantly, *CsPME62* was highly expressed in roots at 4 mM Al treatment, with about 12 and 6.5 fold higher expression than at 0 and 1 mM Al, respectively, implying that this gene may play an important role in the tolerance to Al stress.

## Discussion

### The evolution and characterization of *CsPMEs* in tea plant

It has reported there are 66 *PMEs* in *Arabidopsis* [7], 43 in rice [8], 105 in flax [9], and 80 in *G. arboreum* [10]. We identified 66 *CsPME* genes in the *C. sinensis* genome using the stringent screening criteria. Previous research suggested that during the plant genome evolution, the whole genome duplication (WGD) and tandem repeats played significant roles in the expansion of this gene family [34], thus resulting in various numbers of *PMEs* in different species. Furthermore, evidence indicated that tea plant genome had underwent two rounds of WGD events since they had diverged from the common dicotyledon ancestor. The last event took place between 30 and 40 Mya (millions of years ago), contributing to the widespread genetic recombination that brought about the fifteen chromosomes as we know them in the modern tea plants [35, 36]. After the latest WGD event, almost 50% of the duplicated genes missed one copy whereas the rest diverged promptly via alternatives including subfunctionalization, neofunctionalization, and expression divergence [17]. Thus, unequal distribution of *CsPMEs* on 15 chromosomes in likely a result of gene replication or gene segment replication in evolution history of tea plant genome. Tandem duplication was also a major contributor to the expansion of *PME* gene family [10]. In rice, the *PMEs* family underwent the σ and ρ replication events, with four genes arising from tandem duplication. In *Arabidopsis*, the *PMEs* family went through the α and β copy events, with eight genes being generated via tandem duplication [37]. Our study also revealed that tandem duplication is a predominant driving force contributing to the expansion of *CsPMEs*.

Motif discovery showed that 62.1% of 66 *PME* genes contained all 10 motifs, indicating that the *CsPMEs* were relatively conserved. Eight type-2 *CsPMEs* (proteins with only the PME domains) were shared with motifs 3 and 4, but lacked motif 8. These findings imply that these motifs probably were important in the functional diversity of *CsPMEs*. The structural discrepancy of gene sequences has played a crucial part in the gene family evolution, which is an acclimatization process in speciation and leads to the effective utilization of natural resources or adjustment to stresses/disturbances [38]. The analysis of *CsPME* gene structures indicated that the majority of *CsPMEs* contained two to four exons, whereas *CsPME25* o had a large number (15) of exons. This might have been due to the directional evolution selection of specific *PME* genes in tea plant.

Based on the sequence characteristics and phylogenetic relatedness, we identified 421 *PMEs* from tea plant, black cottonwood, coffee, cacao, kiwi fruit, grapevine, *Arabidopsis*, and rice. The constructed phylogeny tree divided them into seven subgroups. The tea *PMEs* tended to be more corelated to kiwifruit proteins than those from other species. These findings echoed earlier research that suggested kiwifruit is the closest relative of *C. sinensis*. Before the two major varieties *C. sinensis* var.* sinensis*(CSS) and *C. sinensis* var. *assamica* (CSA) diverged from a common ancestor ∼0.38 to 1.54 Mya, the *C. sinensis* diverged from a shared lineage with kiwifruit about 80 Mya [36]. A comprehensive phylogenetic research of 127 plant, fungi and bacteria proteins elucidated that plant *PMEs* are apparently different from the fungi and bacteria subgroups [39]. It was also worthy of noting that there was a clear separation between the type-1 *PMEs* and type-2 *PMEs*, with the last two subfamilies were confined almost completely to type-2 *PMEs*, similarly to the results reported for the cotton [10] and *Arabidopsis PMEs* [40]. In *Arabidopsis*, *P. pilosa* and rice, type-2 *PMEs* account for 65, 57 and 54% of the total *PMEs*, respectively. In tea plant, 12% were identified as type-2 *PMEs*. Based on these results, we presumed that either PME or PMEI domain occurred comparatively late in the evolutionary history, with type-2 *PMEs* probably arising after the divergence of vascular and mosses.

In the evolution analysis, it is of great importance to know the rate of non-synonymous to synonymous mutations (Ka/Ks). In general, non-synonymous substitutions influence the composition of the protein, leading to variations in the conformation and function of proteins, thus resulting in adaptive changes that bring about advantages or disadvantages in the natural selection. By contrast, synonym mutations do not alter the protein components and are therefore unaffected by natural selection. It is generally acknowledged that Ka/Ks value < 1, =1 and > 1 suggest purifying selection, neutral evolution, and positive selection, respectively [41]. The Ka/Ks value of all the homologous *PME* gene pairs in tea plant were < 1, indicating that the duplicated *CsPMEs* had experienced purifying selection.

### *CsPMEs* involved in the growth and development of tea plant

As signal molecules, phytohormones can regulate important life metabolism underpinning plant growth and development at low concentrations [42]. *PMEs* accelerate the de-methylesterified of homogalacturonan that is synthesized in a hypermethylated form before secretion. The extent of homogalacturonan methylesterification plays a decisive role in the structure and function of pectin [43]. In *Arabidopsis*, application of external GA contributed to increased l esterification of HG, indicating a GA requirement for functioning of *PMEs* [44, 45]. Here, we analyzed the promoter regions of 66 *CsPMEs*, and showed that many cis-regulatory elements were related to plant hormones, such as MeJA, GA, ABA and SA. Further studies should characterize the connections between these phytohormones and *CsPMEs.*

Substantial attention has been paid to the function of *PMEs* in plant growth and development. In *Arabidopsis*, de-methylesterification of pectin in the primary cell walls by *PME35* is indispensable for offering a mechanically efficient support to the stem [40], whereas *PME58* (via HG modification) influenced the distribution of pectin in time of seed coat mucilage exudation [46]. In flax (*Linum usitatissimum*) [9] and cotton (*G. arboreum*) [10], some of the *PME* gene family members were highly expressed in the stage of secondary wall thickening (associated with fiber accumulation). By contrast, our results showed a unique expression pattern, as some of the *CsPME* genes were highly significantly expressed only in specific tissues. For example, *CsPME39, CsPME40* and *CsPME41* were highly expressed in roots, *CsPME33, CsPME34* and *CsPME35* in apical bud, and *CsPME51* in young leaves. Taken together, our findings implied that the *CsPME* family genes may perform diverse functions in the growth and development of tea plant. The in-depth roles of *CsPME* genes need to be characterized in further studies.

### *CsPME*s are involved in Al stress responses of tea plant

Previous work has shown that the higher the degree of pectin methylation, the weaker the Al binding capacity of pectin. This results in a decreased amount of Al entering the root system, which is equivalent to the exclusion of Al from the root system, indirectly improving Al resistance of plants. In our study, we observed identical trends for Al content and PME activity variation under in three Al treatments, suggesting that PME participated in the absorption and accumulation of Al in tea plant. In maize, there was a negative connection between the degree of methylesterification of pectin in the suspension cells and Al tolerance [11, 12]. In rice, six *PME* genes were up-regulated in the 50 μM Al treatment, however, in transgenic rice overexpressing one of these six genes (*OsPME14*), the PME activity and Al content in the cell wall of root tips were both enhanced, leading to a decrease in resistance to Al stress [13, 14]. In rye (*Secale cereale*), a *PME* gene was isolated and characterized, the expression of this *ScPME* gene was prominently suppressed by Al in the roots of the tolerant cultivar [6]. These results adequately demonstrated the role of *PMEs* in regulating plant Al resistance.

In tea plant, as an Al hyperaccumulator plant, the major Al detoxification mechanism was binding of Al in the cell wall [2]. Li [15] observed the increased expression of *CsPME* genes was followed by a decreased level of esterified pectins in the Al treatments. Here, we analyzed the expression of 66 *CsPME* genes in in roots and leaves of plants grown in the 0, 1 and 4 mM Al treatments. Results indicated that the expression of *CsPMEs* showed a tissue specific pattern in different Al treatments. Importantly, the expression of eight genes (*CsPME12*, *CsPME13*, *CsPME14*, *CsPME29*, *CsPME6*3, *CsPME64*, *CsPME65*, and *CsPME66*) in leaves and 15 genes (*CsPME1*, *CsPME2*, *CsPME30*, *CsPME3*9, *CsPME40*, *CsPME42*, *CsPME45*, *CsPME47*, *CsPME49*, *CsPME53*, *CsPME54*, *CsPME55*, *CsPME56*, *CsPME59*, and *CsPME60*) in roots displayed the same trend as the variation in Al contents and PME activities in different Al treatments. Molecular function of these genes needs to be elucidated in the future research.

## Conclusion

In this study, we performed a systematic analysis of the *PME* gene family in tea plant. Sixty-six *CsPME* genes were identified and divided into three subfamilies. The motifs, intron-exon distributions, cis-elements features, and evolutionary relationships with seven plant species were investigated. Finally, the tissue expression patterns of *CsPME* genes, as well as their transcription levels were analyzed in the Al treatments. Our study provided a new direction for further research on the functioning of *PME* gene in Al tolerance of tea plant.

## Supplementary Information

**Additional file 1: Table S1.** CDS and protein sequences of *CsPMEs* identified in this study. **Table S2.** Basic information of *CsPMEs*. **Table S3.** The characteristics of *CsPMEs* promoters. **Table S4.** Ka, Ks, Ka/Ks values for tea PME genes. **Table S5.** Primers used in this study. **Table S6.** The expression level of *CsPMEs* in different tissues. **Table S7.** The expression level of *CsPMEs* under Al stress.

**Additional file 2: Figure S1.** Conserved motif of *CsPMEs*.

## Data Availability

All data generated or analyzed in this study are included in this published article (Additional file 1. xlsx). The genome sequences of tea plant, rice and *Arabidopsis* were downloaded from the http://tpdb.shengxin.ren/, https://www.ncbi.nlm.nih.gov/genome/?term=RICE, and http://www.arabidopsis.org/browse/genefamily/.

## Abbreviations

Al: Aluminum
PME: Pectin methylesterase
HG: Homogalacturonan
PG: Polygalacturonase
PL: Pectinlyase
ICP-OES: Coupled plasma optical emission spectrometer
HMM: Hidden Markov Model
PMEI: Pectin methyl esterase inhibitor
MW: Molecular weight
pI: Theoretical isoelectric point
GRAVY: Grand average of hydropathicity
GSDS: Gene Structure Display Server
Ka/Ks: The ratio of the number of nonsynonymous substitutions per nonsynonymous site (Ka) to the number of synonymous substitutions per synonymous site (Ks)
MeJA: Jasmonic acid methyl ester
ABA: Abscisic acid
SA: Salicylic acid
GA: Gibberellin
ARE: Anaerobic induction element
LTR: Low-temperature responsiveness
WUN-motif: Wound-responsive element
ABRE: ABA response element
AuxRE: Auxin-responsive element
GARE-motif: Gibberellin-responsive element
MRE: MYB binding site involved in light responsiveness
MBSI: MYB binding site involved in flavonoid biosynthetic genes regulation
WGD: Whole genome duplication
Mya: Millions of years ago
