# Supplementary figures and images for: Genome-wide identification of PME gene family and expression of candidate genes associated with aluminum tolerance in tea plant (Camellia sinensis)
Source: BMC Plant Biol. 2022 Jun 24;22:306. doi: 10.1186/s12870-022-03686-7 (PMC9229754; doi:10.1186/s12870-022-03686-7)

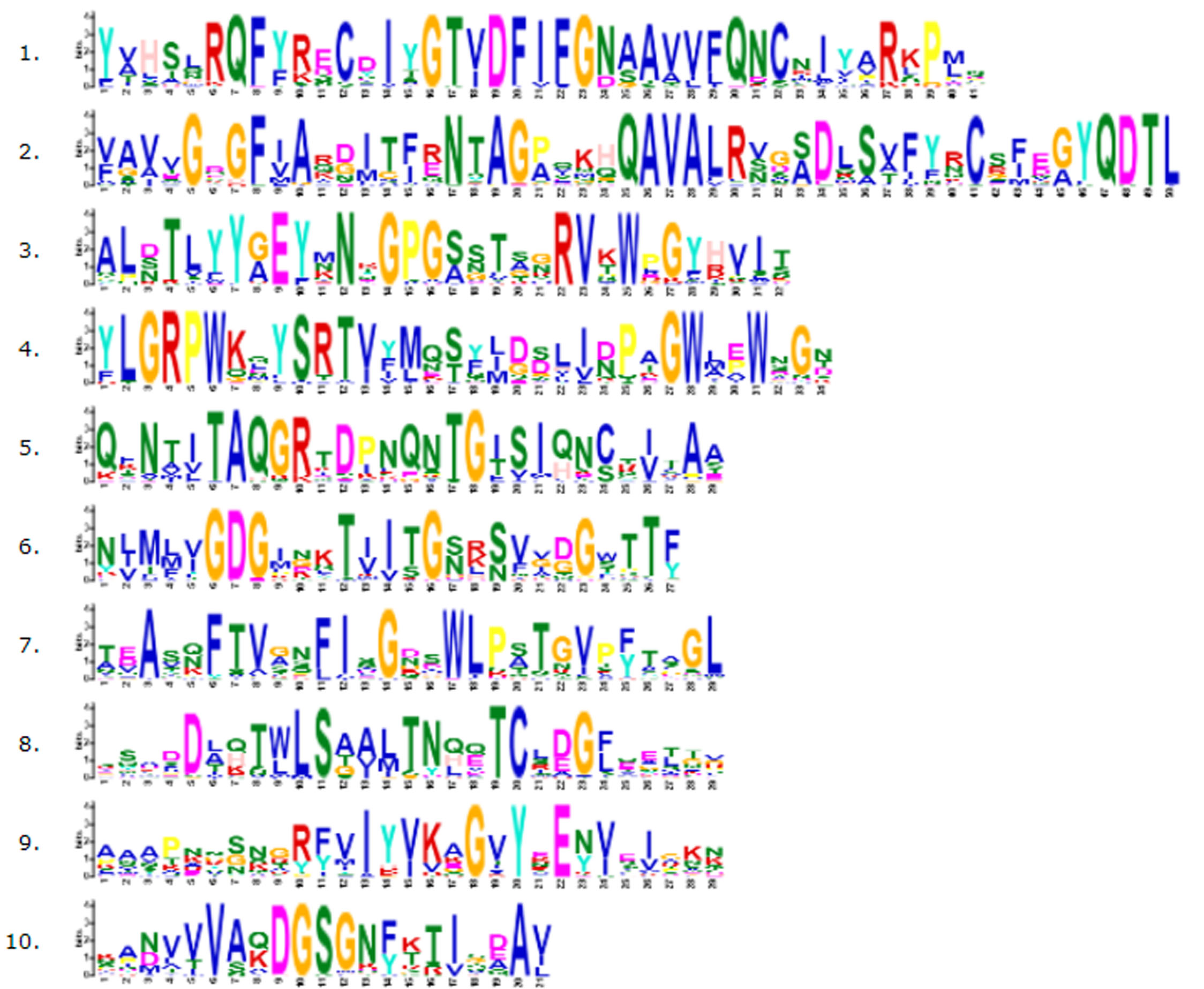

Supplement: Supplementary file 2 — Additional file 2: Figure S1. Conserved motif of CsPMEs. [file 12870_2022_3686_MOESM2_ESM.jpg]
